# Chickpea: Its Origin, Distribution, Nutrition, Benefits, Breeding, and Symbiotic Relationship with *Mesorhizobium* Species

**DOI:** 10.3390/plants13030429

**Published:** 2024-02-01

**Authors:** Junjie Zhang, Jingqi Wang, Cancan Zhu, Raghvendra Pratap Singh, Wenfeng Chen

**Affiliations:** 1 College of Food and Bioengineering, Zhengzhou University of Light Industry, Zhengzhou 450002, China; wjq262023@163.com (J.W.); zcc890986@163.com (C.Z.); 2 Collaborative Innovation Center for Food Production and Safety of Henan Province, Zhengzhou 450002, China; 3 Department of Research and Development, Biotechnology, Uttaranchal University, Dehradun 248007, India; singh.dr.raghvendra@gmail.com; 4 College of Biological Sciences and Rhizobium Research Center, China Agricultural University, Beijing 100193, China

**Keywords:** chickpea, cultivation, rhizobia, breeding, distribution

## Abstract

Chickpea (*Cicer arietinum* L.), encompassing the desi and kabuli varieties, is a beloved pulse crop globally. Its cultivation spans over fifty countries, from the Indian subcontinent and southern Europe to the Middle East, North Africa, the Americas, Australia, and China. With a rich composition of carbohydrates and protein, constituting 80% of its dry seed mass, chickpea is also touted for its numerous health benefits, earning it the title of a ‘functional food’. In the past two decades, research has extensively explored the rhizobial diversity associated with chickpea and its breeding in various countries across Europe, Asia, and Oceania, aiming to understand its impact on the sustainable yield and quality of chickpea crops. To date, four notable species of *Mesorhizobium*—*M. ciceri*, *M. mediterraneum*, *M. muleiense*, and *M. wenxiniae*—have been reported, originally isolated from chickpea root nodules. Other species, such as *M. amorphae*, *M*. *loti*, *M*. *tianshanense*, *M*. *oportunistum*, *M*. *abyssinicae*, and *M. shonense*, have been identified as potential symbionts of chickpea, possibly acquiring symbiotic genes through lateral gene transfer. While *M. ciceri* and *M. mediterraneum* are widely distributed and studied across chickpea-growing regions, they remain absent in China, where *M. muleiense* and *M. wenxiniae* are the sole rhizobial species associated with chickpea. The geographic distribution of chickpea rhizobia is believed to be influenced by factors such as genetic characteristics, competitiveness, evolutionary adaptation to local soil conditions, and compatibility with native soil microbes. Inoculating chickpea with suitable rhizobial strains is crucial when introducing the crop to new regions lacking indigenous chickpea rhizobia. The introduction of a novel chickpea variety, coupled with the effective use of rhizobia for inoculation, offers the potential not only to boost the yield and seed quality of chickpeas, but also to enhance crop productivity within rotation and intercropped systems involving chickpea and other crops. Consequently, this advancement holds the promise to drive forward the cause of sustainable agriculture on a global scale.

## 1. Rationale of Chickpea

### 1.1. The Origin of Chickpea, Types, and Distribution

Chickpea, scientifically known as *Cicer arietinum* L., is a member of the Cicereae tribe within the Fabaceae family, specifically belonging to the Papilionaceae subfamily [1]. This ancient pulse crop holds significant importance as one of the world’s most vital legume crops [2,3]. Across various cultures and languages, chickpea is embraced with a plethora of names, such as garbanzo in Spanish, pois chiche in French, kichar or chicher in German, chana in Hindi, and gram or Bengal gram in English [1]. Its global presence is further reflected in its Turkish name ‘nakhut’ and Romanian, Bulgarian, Afghan, and Russian name ‘nohut’ [4]. Archaeological excavations in Middle Eastern countries, the birthplace of wild chickpeas like *C. judaicum* Boiss., *C. pinnatifidum* Jaub et Spach, and *C. bijugum* Rech, have uncovered carbonized chickpea seeds. Moreover, two additional wild chickpea species, *C. echinospermum* Davis and *C. reticulatum* Ladiz [2], were discovered in South-East Turkey, further enriching the diversity of this remarkable legume.

Desi and Kabuli represent two widely cultivated chickpea cultivars worldwide. Morphologically distinct, Desi (microsperma) is characterized by its pink flowers and a seed coat that is both colored and notably thick. Conversely, Kabuli (macrosperma) features white flowers and seeds that are either white or beige, bearing a distinct ram’s head shape, thin seed coat, and a seed surface that is smooth to the touch [5]. In India, there is also recognition of an intermediate type known for its pea-shaped seeds of local significance [6]. Seed weights vary, with the Desi cultivar weight ranging from 0.1 to 0.3 g and Kabuli types weighing between 0.2 and 0.6 g. Approximately 80–85% of chickpeas planted in Asia and Africa belong to the Desi cultivar, while in West Asia, North Africa, North America, and Europe, the Kabuli cultivar holds greater prominence [6].

Chickpea cultivation spans over 50 countries, encompassing the Indian subcontinent, North Africa, the Middle East, southern Europe, the Americas, and Australia [6]. On a global scale, it stands as one of the most widely grown pulses, with impressive production figures of 14.2 million tons and an average yield of 0.96 tons per hectare (FAO, 2014). As an affordable protein source, chickpea holds immense significance for low-income consumers worldwide, especially in developing nations where access to animal-based protein is limited for large segments of the population [7]. Remarkably, chickpea demonstrates resilience in regions characterized by climate variability, drought, and limited fertilizer usage, which often lead to reduced agricultural productivity [8]. India takes the lead in chickpea production, followed by Pakistan, Turkey, Australia, Myanmar, Ethiopia, Iran, Mexico, Canada, and the USA as other notable contributors [6]. Worth mentioning is China’s emergence as an Asian chickpea producer, with Xinjiang Province in Northwest China as the primary planting hub [9].

### 1.2. Nutritional Quality of Chickpea Seeds

Chickpea is an excellent source of both carbohydrates and protein, which account for 80% of the total dry mass of chickpea [10]. Chickpea has been and continues to be consumed by humans since ancient times owing to its good nutritional properties [6]. It is used as food in different styles in different countries [11], such as chickpea four for making snacks in India [12], and chickpea is used in stews and soups/salads in Asia and Africa [13]. The diverse cooking styles make chickpea appeal to consumers worldwide [6].

#### 1.2.1. Classification of Carbohydrates

Chickpea stands out for its rich carbohydrate content, specifically monosaccharides like ribose and glucose, disaccharides such as sucrose, and oligosaccharides including stachyose and ciceritol [14]. Studies reveal that glucose concentrations in chickpea are 0.7% (*w*/*w*) [15], with maltose and sucrose being the most prevalent free disaccharides [16]. Notably, α-galactosides are the second most abundant carbohydrates in chickpea [14,17], comprising two significant groups: the raffinose family of oligosaccharides (RFOs), which includes raffinose, stachyose, and verbascose [14], and galactosyl cyclitols, with ciceritol being a prime example [18]. Reports indicate that ciceritol and stachyose make up 36–43% and 25% of total sugars, respectively, in chickpea seeds [15,19]. Interestingly, chickpea contains lower levels of absolute flatulent α-galactosides compared to other legume seeds, such as white beans, lentils, or pinto beans [16].

Polysaccharides, i.e., high-molecular-weight monosaccharide polymers, are found in chickpea as storage carbohydrates (like starch) [16] or structural carbohydrates (like cellulose) [10], providing structural support. Starch synthesis and storage are primary functions of chickpea, accounting for the majority of carbon storage in pulse seeds. The starch content in chickpea ranges from 41 to 50% of total carbohydrates [20,21,22], with the kabuli variety containing more soluble sugars than the desi type [22]. Notably, chickpea seeds boast a high starch content of approximately 525 g/kg dry matter, with 35% being resistant starch (RS) and the remaining 65% as available starch [19,23]. The in vitro starch digestibility values (ISDVs) of chickpea range between 37% and 60% [24,25], which is relatively high compared to other pulses [26].

#### 1.2.2. Dietary Fiber and Protein Content

Dietary fiber (DF), encompassing both soluble and insoluble forms, represents the portion of plant food that remains undigested in the human small intestine. Chickpea stands out for its high DF content, ranging from 18 to 22 g per 100 g [19,27]. Specifically, raw chickpea seeds contain approximately 4–8 g of soluble DF and 10–18 g of insoluble DF per 100 g [20,28]. However, compared to other legumes, the fiber content of chickpea hulls on a dry weight basis is lower, at 75%, than that of lentils (87%) and peas (89%) [20].

Interestingly, the desi variety of chickpea boasts a higher content of total DF and insoluble DF compared to the kabuli variety. This difference can be attributed to the thicker hulls and seed coat in desi, which account for 11.5% of the total seed weight, compared with only 4.3–4.4% in kabuli. This variation underscores the diverse characteristics and nutritional profiles within the chickpea family, making it a versatile and valuable addition to a balanced diet [28].

Chickpea stands out for its protein content, which can range from 17–22% of the dry seed [29,30]. Notably, the quality of chickpea protein is superior to that of some other pulse crops, such as *Vigna mungo* L. [31], *Vigna radiata* L., and *Cajanus cajan* L. However, variations in crude protein concentration exist between the kabuli [K] and desi [D] types, with some studies reporting significant differences (241 g Kg^−1^ in K versus 217 g kg^−1^ in D) while others do not show such disparities (217 g kg^−1^ in K versus 215 g kg^−1^ in D) [28]. This inconsistency highlights the importance of considering specific varieties and growing conditions when evaluating chickpea’s nutritional profile.

Furthermore, chickpea seeds contain a total of 18 different amino acids, emphasizing their nutritional completeness [32,33,34]. Interestingly, there are no significant differences in amino acid contents between the kabuli and desi varieties [32,33], indicating that both types offer similar benefits in terms of protein quality and amino acid composition. This consistency in amino acid profiles among chickpea varieties adds to their value as a reliable source of plant-based protein, suitable for various dietary needs and preferences.

#### 1.2.3. Fatty Acid Profile

Wood and Grusak [16] reported a fat content ranging from 3.40–8.83% in kabuli and 2.90–7.42% in desi chickpea varieties, surpassing other pulses like lentils, red kidney beans, mung beans, and pigeon peas, as well as wheat and rice (http://www.nal.usda.gov/fnic/foodcomp/search/). Chickpea fatty acid composition consists of approximately 66% polyunsaturated fatty acids (PUFA), 19% monounsaturated fatty acids, and 15% saturated fatty acids. Notably, kabuli types tend to have higher amounts of oleic acid, while desi types have higher amounts of linoleic acid. Chickpea stands out as a rich source of nutritionally essential PUFA, specifically linoleic acid (51.2%; LA) and monounsaturated oleic acid (32.6%; OA), outpacing other edible pulses [32]. Additionally, chickpea’s fatty acid profile is predominantly linoleic acid, followed by oleic and palmitic acids.

#### 1.2.4. Minerals and Vitamins

Chickpea offers consumers a plethora of essential vitamins and minerals [35,36], including iron, zinc, magnesium, and calcium [35]. Selenium is also present in chickpea seeds, making it an even more valuable addition to a balanced diet (http://www.nal.usda.gov/fnic/foodcomp/search/) [35]. A mere 100 g of raw chickpea seeds can provide approximately 5.0 mg of iron, 4.1 mg of zinc, 138 mg of magnesium, and 160 mg of calcium per 100 g. Impressively, just 100 g of chickpea seeds can fulfill the daily dietary requirements for iron (1.05 mg/day for males and 1.46 mg/day for females) and zinc (4.2 mg/day and 3.0 mg/day), while 200 g can meet magnesium needs (260 mg/day and 220 mg/day) (FAO 2002). Interestingly, there are no significant differences in mineral content between the kabuli and desi varieties, except for calcium [32,37]. Additionally, chickpea contains various elements such as aluminum, chromium, nickel, lead, and cadmium [38]. It is also rich in folic acid [39] and provides moderate amounts of water-soluble vitamins like riboflavin (B2), pantothenic acid (B5), and pyridoxine (B6) [40].

### 1.3. Health Benefits of Chickpea

Chickpea’s consumption offers various physiological perks, making it a potential candidate for the classification of ‘functional food’, beyond its widely recognized role in protein and fiber provision [6].

#### 1.3.1. Diabetes and Blood Pressure

Chickpeas boast a high content of resistant starch and amylose [41]. With a greater degree of polymerization, amylose offers increased resistance to digestion in the small intestine, thus leading to a slower conversion into glucose [41,42]. This results in a delayed glucose entrance into the bloodstream, subsequently reducing insulin requirements, lowering the glycemic index (GI), and mitigating the insulinemic postprandial response [43,44]. These collective effects are instrumental in decreasing both the occurrence and intensity of type II diabetes [45]. Moreover, linoleic acid plays a pivotal role in prostaglandin production, which is crucial in regulating blood pressure levels [46].

#### 1.3.2. Reduce the Risk of Cancer

Butyrate is known to hinder cell proliferation [47] and trigger apoptosis [48], collectively diminishing the likelihood of colorectal cancer. Prior studies indicate that incorporating β-sitosterol (chickpea’s primary phytosterol) in a rat’s diet can curtail the development of colonic tumors induced by N-methyl-N-nitrosourea (a carcinogen) [49]. Moreover, the presence of lycopene in chickpea seeds may offer protection against prostate cancer [50]. In a different investigation, an extract of chickpea isoflavones was discovered to impede epithelial tumor growth while leaving healthy cells unaffected [51].

#### 1.3.3. Control of Weight

Chickpea supplementation has been observed to hinder the escalation of body weight and epididymal adipose tissue mass in rats [52]. Over the course of the experiment, rats fed a high-fat diet (HFD) reached 654 g, whereas those given an HFD with chickpea supplementation (HFD + CP) weighed 562 g. Notably, the ratio of epididymal fat pad weight to total body weight was considerably higher in rats on a pure HFD (0.032 g/g) compared to those on HFD + CP (0.023 g/g) [52]. This underscores chickpea’s potential as a low-GI food that could be a valuable addition to weight loss regimens. Additionally, chickpea consumption has been found to mitigate fat accumulation in individuals with obesity. Incorporating chickpea into the diet leads to increased feelings of satiation and fullness [53]. In a study, forty-two participants adopted a chickpea-enriched diet (averaging 104 g/day) for twelve weeks, sandwiching this period with their usual diet for four weeks each [53].

#### 1.3.4. Gut Health

In an experiment with 19 healthy persons, there was a significant increase (18%) in DF through the consumption of 140 g/day chickpea and chickpea flour for six weeks [54]. Also, in the test conducted by Murty et al. [53], there was an overall improvement in bowel health such as an increased frequency of defecation, ease of defecation, and softer stool consistency in those fed a chickpea diet compared to a habitual diet. The DF played a positive role in promoting bowel function [6].

### 1.4. Breeding of Chickpea

Globally, chickpea cultivation spans over 13.2 million hectares, with an annual yield of 13.1 million tons, yet its productivity remains below 1 ton per hectare, despite having the potential to reach 6 tons per hectare [55]. The primary challenges to chickpea production encompass both biotic (such as Helicoverpa, Bruchus, Aphidoidea, and Ascochyta) and abiotic (including drought, heat, salt, and cold) stresses, leading to a 10% decrease in yield [56]. However, to boost chickpea productivity, it is imperative to tackle these biotic and abiotic stresses [57]. Consequently, chickpea breeders worldwide are concentrating on developing cultivars with multiple resistances to both biotic and abiotic factors [58]. Additionally, advanced chickpea genotypes with exceptional yield have been developed through the integration of genes offering resistance to drought, cold, salinity, fungi, and pod borers [59]. A range of strategies, including traditional breeding methods, molecular breeding, and modern plant breeding techniques, are being employed to address these challenges [56].

Breeders have extensively utilized conventional technologies such as introduction, selection, hybridization, and mutation [60]. Techniques involving single, multiple, and three-way crosses have been employed in breeding chickpeas [61,62]. Apart from these conventional methods, novel technologies like genetic modification have been utilized to cultivate chickpeas with desirable traits. Additionally, biotechnological techniques have been employed to develop transgenic chickpeas with enhanced resistance to multiple stresses [56]. Worldwide, over 100 gene banks maintain approximately 100,000 chickpea accessions [63], offering breeders valuable genetic resources to develop improved chickpea varieties [57]. However, a sequence of ‘genetic bottleneck’ events has led to a limited genetic base for cultivated chickpeas [64,65].

The enhancement of genetic traits, whether through traditional or molecular techniques, faces constraints not only due to limited genomic resources but also because of the narrow genetic diversity within the elite gene pool [66]. To elucidate the genetic foundations of various agronomic characteristics for yield enhancement at the molecular level, having an accurate chickpea genome assembly is paramount for both fundamental and applied research [67]. Over the past decade, the widespread adoption of NGS (Next-Generation Sequencing) technology has transformed chickpea from an orphan crop to one with abundant genetic, genomic, and transcriptomic resources [68,69,70,71,72,73,74]. To gain novel insights into genome organization, evolution, domestication, and genetic diversity and to expedite molecular breeding for enriching chickpea’s genetic traits, the initial whole-genome shotgun sequencing of the kabuli chickpea cultivar was completed [75]. Subsequently, by analyzing the WGS (Whole Genome Sequencing) data of 3366 chickpea germplasm accessions, a comprehensive map of genetic variations in chickpea was reported, which in turn suggested three genomic breeding strategies for chickpea [76].

To expedite agricultural advancement, innovative crop enhancement practices are continuously emerging worldwide [77]. For instance, chickpeas with stress resilience have been developed through transgenic technology and genome editing techniques. In research on chickpea’s biological resistance, transgenic chickpeas containing α-AI1 demonstrated the inhibition of *Callosobruchus maculatus* and *C. chinensis* in insect tests [78]. In another study, chickpeas resistant to aphids were created through the expression of a novel insecticidal lectin [79]. Moreover, transgenic breeding programs have yielded chickpeas expressing chimeric *cry1Aabc*, effective against the gram pod borer [80]. Although numerous chickpea varieties resistant to *H. armigera* have been developed in India, only a few have been successfully implemented in agricultural settings [81]. Regarding abiotic stress, research indicates that the *DREB1A* transcription factor enhances root and shoot growth, leading to improved transpiration efficiency, thereby maintaining drought resistance in transgenic chickpeas [82]. Additionally, chickpeas harboring the *P5CS* gene exhibit strong salt tolerance [83]. To cultivate chickpea varieties with enhanced abiotic stress resistance, further research in molecular breeding, particularly gene editing, is essential [84].

## 2. Chickpea Rhizobia and the Inoculation Effects on Chickpea Production

### 2.1. Diversity, Geographic Distribution, and Natural Succession of Chickpea Rhizobia

Throughout the past two decades, the global research community has delved into the vast diversity of chickpea rhizobia, with investigations spanning Europe [85,86], Asia [87], and Oceania [88]. To date, studies have documented the primary isolation of *Mesorhizobium ciceri* [85], *Mesorhizobium mediterraneum* [86], *Mesorhizobium muleiense* [89], and *Mesorhizobium wenxiniae* [90], specifically from the root nodules of chickpeas. Furthermore, research has also uncovered various *Mesorhizobium* species, such as *M. amorphae*, *M. loti*, *M. tianshanense*, *M. oportunistum*, *M. abyssinicae*, and *M. shonense*, as symbiotic partners of chickpeas [91,92,93]. This exploration emphasizes the widespread presence and significance of these rhizobia in the agricultural ecosystem, particularly in relation to chickpea cultivation.

**Figure 1:**
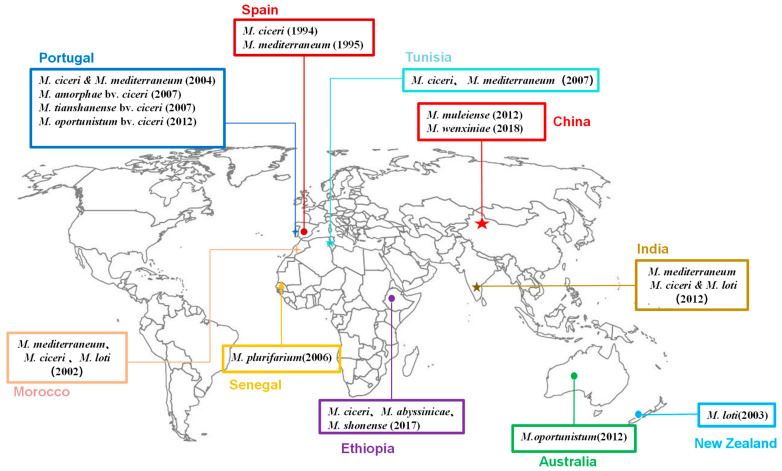
Geographic distribution of rhizobial species associated with chickpea worldwide.

*M. ciceri* and *M. mediterraneum* are widespread across numerous countries, including Spain, Portugal, Morocco, Tunisia, and India (as depicted in Figure 1). However, their presence is conspicuously absent in China, where instead, *M. muleiense* and *M. wenxiniae* have been discovered and identified [87,89,90,94,95]. Notably, *M. muleiense* exhibits a broader distribution in the Xinjiang, Gansu, and Ningxia regions of northwestern China [9,87,89,94,95], whereas *M. wenxiniae* is restricted to Gansu Province [90]. When chickpeas are introduced into a new region lacking native chickpea rhizobia, it becomes imperative to inoculate specific rhizobia during the introduction and planting process [96]. Remarkably, in China, none of the globally prevalent chickpea rhizobia, such as *M. ciceri*, have been detected [9]. This absence suggests a long history of chickpea introduction in China, leading to the emergence of unique Chinese chickpea rhizobial species through the planting process. During this time, rhizobia may have been co-transported with chickpea seeds, potentially allowing native Chinese rhizobia to acquire symbiotic capabilities from introduced chickpea rhizobia or even evolve directly to nodulate with chickpeas through co-evolutionary processes [87,96].

Zhang et al. [9] conducted a pivotal study on the natural evolution of chickpea rhizobia, focusing on *M. muleiense*—the sole chickpea rhizobial species in Xinjiang Province in northwestern China. Over a seven-year period spanning from 2009 to 2016, the research delved into the *recA* genotypes of this species, revealing a total of 28 genotypes, illustrative of the natural succession of *M. muleiense*. Throughout the sampling years and locations, four primary genotypes exhibited consistency, while certain genotypes appeared exclusively in specific years or at specific sites, pointing to a time-geographic succession model. Additionally, despite the consistent presence of these four main genotypes, their quantitative prevalence varied between sampling years, potentially influenced by differences in agricultural practices. Moreover, the study identified soil pH values and the potassium content as key non-biological factors impacting the natural evolution of *M. muleiense*, a distinct species of chickpea rhizobium in China.

### 2.2. Effects of Chickpea Rhizobial Inoculation on Chickpea

Rhizobia are pivotal players in diverse soil biochemical processes, fostering host plant growth and enriching soil quality [97]. The symbiotic alliance between legumes and rhizobia stands as the preeminent N_2_-fixing mechanism in agricultural systems [98]. Additionally, rhizobia possess the capability of phosphate solubilization, thereby enhancing growth in certain legumes [99], and mitigating plant ethylene levels through ACC deaminase activity [100]. Biological nitrogen fixation (BNF) sustains sustainable agriculture by supplementing chemical fertilizers, ensuring optimal crop yields. Under optimal circumstances, this symbiotic N_2_ fixation can meet up to 85% of the nitrogen demands in legumes [101].

#### 2.2.1. Effect of Rhizobial Inoculation on the Composition and Diversity of the Rhizosphere Microorganisms of Chickpea

*M. ciceri* is widely distributed globally, except in China where *M. muleiense* is the primary species associated with chickpea [96]. In the unsterilized soils of Xinjiang, the local species of *M. muleiense* displays stronger competitiveness compared to the introduced *M. ciceri*. However, in sterilized soils, *M. ciceri* outcompetes *M. muleiense* [102]. Notably, *M. ciceri* demonstrates a higher competitive ability in soils from new chickpea cultivation regions compared to *M. muleiense* [102]. The introduction of different rhizobial species can impact the chickpea rhizosphere microbiota in soils from various planting areas. In the rhizosphere of chickpeas grown in Xinjiang’s traditional soils and newly introduced zones, eight dominant phyla with 34 dominant genera and 10 dominant phyla with 47 dominant genera were identified after inoculation with *M. muleiense* and *M. ciceri* rhizobia, respectively. Proteobacteria and Actinobacteria were among the dominant phyla present in all soil rhizospheres. Interestingly, the genus *Pseudomonas* was significantly enriched after inoculation with *M. muleiense* in Xinjiang soils, but not in newer chickpea cultivation areas. This leads to the speculation that *Pseudomonas* could be the key microorganism influencing the competitive nodulation of various chickpea rhizobia in different regions [103].

#### 2.2.2. Effect of Rhizobial Inoculation on Plant Growth

In Rudresh et al.’s study [104], the positive impact of rhizobium inoculation on chickpea growth was evident, with inoculated plants exhibiting greater plant height, branch count, and biomass compared to uninoculated ones. Elkoca et al. [105] also observed increases in plant height, shoot dry weight, and chlorophyll content in chickpeas inoculated with rhizobia. These inoculated treatments also led to longer roots [106], which expanded the root surface area and subsequently boosted nutrient uptake [107]. Shahzad et al.’s findings [108] concurred, highlighting that rhizobial inoculation fostered greater plant height and shoot biomass. Rhizobium inoculation has the potential to enhance the growth and development of photosynthetic organs, subsequently accelerating the accumulation of photosynthates [109]. In our prior research, USDA 3378 inoculation resulted in a substantial increase in chickpea root dry weight (2.82-fold), shoot dry weight (2.62-fold), and chlorophyll content (2.34-fold) compared to the control group [96]. Co-inoculation with USDA 3378 and CCBAU 83963 also yielded promising symbiotic efficiency, albeit with a slight reduction in shoot dry weight, possibly due to strain competition affecting biological nitrogen fixation [96].

#### 2.2.3. Nutrient Content, Uptake, and Protein

Chickpea is prized for its rich protein content, boasting approximately 16–20% protein in its grains, a trait closely linked to its nitrogen content. Boosting nitrogen levels through chickpea rhizobial inoculation can potentially increase the protein content [101]. Research conducted by Erman et al. [110] revealed that grains with enhanced shoot N and P contents outperformed uninoculated controls. Similarly, Kumar et al. [111] observed higher protein levels in plants inoculated with rhizobium. Field experiments have also shown that chickpea plants treated with rhizobial inoculation exhibit increased N and P uptake in both grains and shoots compared to uninoculated controls [112]. This improvement can likely be attributed to augmented nitrogen fixation [112] and enhanced root growth, leading to improved nutrient acquisition [113].

#### 2.2.4. Selection of the Best Strain for the Inoculation of Chickpea in China

Over the last 15 years, numerous farming inoculation trials have taken place across eastern China, spanning Jilin Province in the northeast, Henan Province in the central region, Shandong Province in the east, and Yunnan Province in the southwest. These selected test locations lacked native chickpea rhizobia in their soil, making inoculant production and application necessary. The chosen strains for the trials were the introduced *Mesorhizobium ciceri* USDA 3378^T^ and an indigenous strain, *Mesorhizobium muleiense* CCBAU 83963^T^. Results revealed that the introduced USDA 3378 strain exhibited a notable competitive edge in nodulation, with nodulation rates ranging between 84.6% and 100% in all newly introduced chickpea soils [96]. Chickpea plants inoculated with USDA 3378 exhibited superior symbiotic performance, evident in plant dry weight, leaf chlorophyll content, and nodule count. Moreover, it formed nodules approximately 2 days sooner than CCBAU 83963. Additionally, USDA 3378 demonstrated higher growth rates in media and stronger adsorption abilities on chickpea roots. Consequently, USDA 3378 was chosen as the preferred strain for developing chickpea rhizobial inoculants in China’s newly introduced chickpea regions. This development holds promise for enhancing soil conditioning and fostering environmentally friendly chickpea production in China [96].

## 3. Conclusions

The presented information will unveil the origins, dispersal, and nutritional worth of chickpeas, alongside their pivotal role in bolstering consumers’ wellbeing. Chickpea stands as the second most prominent legume globally, enriched with proteins, carbohydrates, minerals, vitamins, dietary fiber, and fatty acids. Prior research indicates that chickpea components may mitigate the risks of diverse chronic ailments, albeit the underlying mechanisms remain elusive. Breeding chickpeas involves a blend of conventional and modern molecular techniques, enabling scientists to cultivate multi-resistant varieties leveraging prior genomic data for optimized growth in challenging environments. Copious evidence underscores that inoculating chickpeas with rhizobia prior to sowing fosters their growth and enhances symbiotic attributes, nutrient uptake, and overall quality. This is attributed to optimized nutrient acquisition.

To reduce cultivation expenses and enhance chickpea quality, combining rhizobial inoculation with inorganic fertilizers offers a promising solution. This approach is particularly vital when introducing chickpeas to new regions lacking a planting history, as these soils often lack native chickpea rhizobia. Studies reveal that various chickpea rhizobial strains exhibit differing adaptation and competitive nodulation capabilities, with *Pseudomonas* potentially playing a pivotal role in soil biology. Inoculation with high-performance rhizobia not only boosts bio-nitrogen fixation and plant growth but also significantly minimizes chemical nitrogen and pesticide usage, crucial for sustainable agriculture. In conclusion, chickpea’s global significance as a legume is unmistakable, and continuous breeding efforts combined with rhizobial inoculation will undoubtedly benefit chickpea producers, consumers, and agroecologists alike.

## Data Availability

Not applicable.
